# Cross-validation study between the HRRT and the PET component of the SIGNA PET/MRI system with focus on neuroimaging

**DOI:** 10.1186/s40658-020-00349-0

**Published:** 2021-02-26

**Authors:** Julia G. Mannheim, Ju-Chieh (Kevin) Cheng, Nasim Vafai, Elham Shahinfard, Carolyn English, Jessamyn McKenzie, Jing Zhang, Laura Barlow, Vesna Sossi

**Affiliations:** 1 Department of Physics and Astronomy, University of British Columbia, Vancouver, British Columbia Canada; 2 Werner Siemens Imaging Center, Department of Preclinical Imaging and Radiopharmacy, Eberhard-Karls University Tuebingen, Tuebingen, Germany; 3 Cluster of Excellence iFIT (EXC 2180) “Image Guided and Functionally Instructed Tumor Therapies”, University of Tuebingen, Tuebingen, Germany; 4 Pacific Parkinson’s Research Centre, University of British Columbia, Vancouver, British Columbia Canada; 5 Djavad Mowafaghian Centre for Brain Health, Pacific Parkinson’s Research Centre, University of British Columbia & Vancouver Coastal Health, Vancouver, British Columbia Canada; 6 Global MR Applications & Workflow, GE Healthcare Canada, Vancouver, British Columbia Canada; 7 UBC MRI Research Centre, University of British Columbia, Vancouver, British Columbia Canada

**Keywords:** Cross-validation study, HRRT, PET/MR, Recovery coefficients, Binding potentials

## Abstract

**Background:**

The Siemens high-resolution research tomograph (HRRT - a dedicated brain PET scanner) is to this day one of the highest resolution PET scanners; thus, it can serve as useful benchmark when evaluating performance of newer scanners. Here, we report results from a cross-validation study between the HRRT and the whole-body GE SIGNA PET/MR focusing on brain imaging.

Phantom data were acquired to determine recovery coefficients (RCs), % background variability (%BG), and image voxel noise (%). Cross-validation studies were performed with six healthy volunteers using [^11^C]DTBZ, [^11^C]raclopride, and [^18^F]FDG. Line profiles, regional time-activity curves, regional non-displaceable binding potentials (BP_ND_) for [^11^C]DTBZ and [^11^C]raclopride scans, and radioactivity ratios for [^18^F]FDG scans were calculated and compared between the HRRT and the SIGNA PET/MR.

**Results:**

Phantom data showed that the PET/MR images reconstructed with an ordered subset expectation maximization (OSEM) algorithm with time-of-flight (TOF) and TOF + point spread function (PSF) + filter revealed similar RCs for the hot spheres compared to those obtained on the HRRT reconstructed with an ordinary Poisson-OSEM algorithm with PSF and PSF + filter. The PET/MR TOF + PSF reconstruction revealed the highest RCs for all hot spheres. Image voxel noise of the PET/MR system was significantly lower. Line profiles revealed excellent spatial agreement between the two systems. BP_ND_ values revealed variability of less than 10% for the [^11^C]DTBZ scans and 19% for [^11^C]raclopride (based on one subject only). Mean [^18^F]FDG ratios to pons showed less than 12% differences.

**Conclusions:**

These results demonstrated comparable performances of the two systems in terms of RCs with lower voxel-level noise (%) present in the PET/MR system. Comparison of in vivo human data confirmed the comparability of the two systems. The whole-body GE SIGNA PET/MR system is well suited for high-resolution brain imaging as no significant performance degradation was found compared to that of the reference standard HRRT.

## Background

Positron emission tomography (PET) is one of the most sensitive non-invasive in vivo imaging techniques [1] and has demonstrated its tremendous impact in clinical and research studies [2–5].

The high resolution research tomograph (HRRT, CTI PET Systems, Knoxville, TN, USA), introduced in the late 1990s/early 2000s, is to this day arguably one of the highest resolution PET scanners for human brain imaging [6]. The double layer of cerium-doped lutetium oxyorthosilicate (LSO) and cerium-doped lutetium-yttrium oxyorthosilicate (LYSO) crystals, which enable photon depth-of-interaction (DOI) detection, results in a spatial resolution of (~ 2.5 mm)^3^ at 1 cm tangential offset from center of the field of view (cFOV) and fairly uniform across the FOV [7]; the scanner can thus serve as a very useful benchmark when evaluating performance of newer scanners. Given that the HRRT is no longer commercially available, most dedicated brain imaging sites consider acquiring newer PET systems. Even though in most cases dedicated brain PET scanners would be preferred for cost, and potentially better sensitivity and resolution performance, the choice is generally practically limited to whole-body hybrid systems, i.e., whole-body PET systems in combination with computed tomography (CT) or magnetic resonance (MR) imaging, as standalone dedicated PET systems such as the HRRT are no longer available or common.

The GE SIGNA PET/MR system (GE Healthcare, Chicago, IL, USA) is the first whole-body hybrid PET/MR system based on silicon photomultipliers (SiPMs) with time-of-flight (TOF) capabilities. The PET detectors are integrated in the MR bore allowing whole-body simultaneous acquisition of PET and MR [8] with high PET detection stability [9].

The aim of this work was to perform a cross-validation study between the HRRT and the SIGNA PET/MR beyond the National Electrical Manufacturers Association (NEMA) evaluation to provide a direct and clinically relevant comparison with particular focus on brain imaging. While the benefits of simultaneous PET/MR imaging for neurological applications have been discussed elsewhere [10, 11], the present study focuses on the comparison of the image quality obtained with TOF data acquired on the PET/MR (default acquisition mode) and the non-TOF data acquired on the HRRT (by hardware default).

In a first step, phantom data were acquired to determine contrast recovery coefficients (RCs) and percent background variability (%BG), as well as percent voxel-level noise (%) for both systems. Cross-validation studies were then performed with six healthy volunteers scanned on both systems with commonly used PET tracers: [^11^C]dihydrotetrabenazine ([^11^C]DTBZ), a marker for the vesicular monoamine transporter 2 (VMAT2), the glucose analog [^18^F]-fluorodeoxyglucose ([^18^F]FDG) and [^11^C]raclopride, a D2/3 receptor antagonist. The choice of tracers was dictated by the requirement to examine cases of widespread tracer distribution ([^18^F]FDG) and tracers with more localized distribution and a wide range of count-rates ([^11^C]DTBZ and [^11^C]raclopride).

## Methods

### Systems description

The HRRT system is based on a dual-layer design of 2.44 × 2.44 × 10 mm^3^ LSO/LYSO crystals enabling DOI encoding and coupled to photomultiplier tubes (PMTs). The FOV spans 25 cm in the axial and 35 cm in the transaxial direction [7, 12].

The PET component of the SIGNA PET/MRI is based on SiPMs coupled to 4.0 × 5.3 × 25 mm^3^ lutetium-based scintillation crystals, which enable the use of TOF information during data reconstruction (timing resolution < 400 ps) [8]. The detector ring, spanning an axial FOV of 25 cm and a transaxial FOV of 60 cm [8, 13], is fully enclosed in the MRI scanner. The MR component is equipped with a radiofrequency transmit body coil embedded in a static 3 T magnet. All MR subject data used in this study were acquired with the GE head neck unit 12 channel coil. Table 1 lists a detailed comparison of both systems’ specifications and reported performance parameters.

**Table 1:** System specification and performance parameters of the Siemens HRRT and GE SIGNA PET/MR system

	HRRT	SIGNA PET/MR
**Crystal material**	LSO/LYSO	Lutetium based
**Crystal dimensions [mm**^**3**^**]**	2.44 × 2.44 × 10	4.0 × 5.3 × 25
**Number of crystals**	119,808	20,160
**Detector**	PMTs	Si-PM
**FOV (axial vs. transaxial) [cm]**	25.2 x 31.2	25 × 60
**Reported resolution (radial** × **tangential** × **axial) [mm]**	2.3 × 2.3 × 2.5^a^ [7, 12]	3.48 × 3.43 × 4.67^b^ [8]
**Reported sensitivity**	2.9%^c^ [12]	23.3 cps/kBq^d^ [8]
**Special characteristics**	DOI correction	TOF (timing resolution < 400 ps), simultaneous PET/MRI

^a^At 1 cm tangential offset to the center of the FOV (reconstructed with a 3D ordinary Poisson-ordered subset expectation-maximization algorithm (OP-OSEM))

^b^At 1 cm tangential offset to the center of the FOV (reconstructed with a TOF OSEM algorithm with filter)

^c^At the center FOV based on the NEMA 2001 evaluation protocol; note that measured activity was normalized by the line source length in the scanner FOV (25 cm), rather than its entire length (70 cm)

^d^At the center FOV based on the NEMA 2012 evaluation protocol. Sensitivity of 23.3 cps/kBq is equivalent to 2.3%. When expressed in the same units as the sensitivity measured for the HRRT, the GE SIGNA sensitivity is estimated to be 6.5%

### Phantom studies

RCs and %BG variability were determined using a cylindrical phantom (referred to as contrast phantom, Flanged Jaszczak ECT Phantom, Data Spectrum Corporation, Durham, NC, USA) scanned on the HRRT and on the PET/MR to enable a direct comparison, as the NEMA recommended phantom does not fit in the HRRT FOV. However, to enable a comparison with other systems, the NEMA recommended image quality phantom was additionally scanned on the PET/MR. Results can be found in the supplementary data.

The contrast phantom contained 6 fillable hollow spheres with inner diameters (IDs) of 9.9, 12.4, 15.4, 19.8, 24.8, and 31.3 mm. The two largest spheres were filled with water; the four smaller spheres and the background (volume 6000 ml) were filled with ^18^F activity in a 3.88:1 and 3.91:1 contrast ratio for the PET/MR and HRRT, respectively (total activity: ~ 29 MBq). No background activity was placed outside the FOV. List-mode data were acquired and histogrammed into one frame with activity concentration and counts matched between scanners.

A transmission scan using a rotating ^137^Cs source was performed on the HRRT to correct for attenuation. HRRT data were reconstructed using an ordinary Poisson-ordered subset expectation maximization (OP-OSEM) algorithm [14] with a 256 × 256 × 207 matrix resulting in a reconstructed voxel size of 1.22 × 1.22 × 1.22 mm^3^. Sixteen subsets and 6 iterations were used for reconstructions without post-filtering (this will be denoted as “native”) and with a 2-mm FWHM Gaussian filter (standard in-house reconstruction), and 10 iterations for reconstructions with PSF [15, 16] and with PSF and a 2-mm FWHM Gaussian filter, respectively (see Table 2).

**Table 2:** Reconstruction parameters used for the HRRT phantom and healthy subject scans

HRRT	Matrix size	Voxel size [mm^3^]	Iterations		Subsets	PSF correction	Filter
			Phantom data	Healthy subject data			
Native	256 × 256 × 207	1.22 × 1.22 × 1.22	1 up to 10	6	16	None	None
Filter	256 × 256 × 207	1.22 × 1.22 × 1.22	1 up to 10	6	16	None	2 mm FWHM gaussian in all 3 dimensions
PSF	256 × 256 × 207	1.22 × 1.22 × 1.22	1 up to 10	10	16	Yes (see reference to PSF correction [15])	None
PSF + filter	256 × 256 × 207	1.22 × 1.22 × 1.22	1 up to 10	10	16	Yes (see reference to PSF correction [15])	2 mm FWHM gaussian in all 3 dimensions

In order to correct the data acquired on the PET/MR for attenuation, the transmission maps acquired on the HRRT were resliced and co-registered to the non-attenuation corrected PET/MR images. The manufacturer’s attenuation map of the PET/MR coil was then co-registered and integrated into the resliced HRRT attenuation map.

PET/MR data were reconstructed using a TOF-OSEM algorithm with 28 subsets, 2 iterations, and a 128 × 128 × 89 matrix resulting in a reconstructed voxel size of 2.781 × 2.781 × 2.780 mm^3^ (reconstructed in-plane FOV: 35.6 cm, GE recommendation for phantom data). Additionally, the data were reconstructed using TOF-OSEM + Gaussian post-filtering with a 3.5 mm full width at half maximum (FWHM) filter in all three dimensions (TOF + filter), TOF-OSEM + resolution modeling with point spread function (PSF, TOF + PSF) (PSF correction was implemented by the manufacturer following the approach from [17]) and TOF-OSEM + PSF + Gaussian post-filtering with a 3.5-mm FWHM filter in all three dimensions (TOF + PSF + filter, Table 3). Manufacturer supplied corrections for decay, random and scattered coincidences, normalization, and dead time were applied. PET/MR phantom data were also reconstructed without TOF information (woTOF) using the same parameter settings as previously described. Different sized post filters between the HRRT and PET/MR were applied to achieve noise reduction without significantly degrading the resolution; i.e., the FWHM of the filter is in each case slightly smaller than the smallest dimension of the detector crystal.

**Table 3:** Reconstruction parameters used for the PET/MR phantom and healthy subject scans

PET/MR	Matrix size		In-plane FOV [cm]	Voxel size [mm^3^]		Iterations		Subsets	PSF correction	Filter	
	Phantom data	Healthy subject data		Phantom data	Healthy subject data	Phantom data	Healthy subject data			Phantom data	Healthy subject data
TOF	128 × 128 × 89	256 × 256 × 89	35.6	2.781 × 2.781 × 2.780	1.391 × 1.391 × 2.780	1 up to 10	4	28	None	None	None
TOF + filter	128 × 128 × 89	256 × 256 × 89	35.6	2.781 × 2.781 × 2.780	1.391 × 1.391 × 2.780	1 up to 10	4	28	None	3.5 mm FWHM gaussian in all 3 dimensions	3.5 mm Gaussian transaxial and 3-point axial convolution filter
TOF + PSF	128 × 128 × 89	256 × 256 × 89	35.6	2.781 × 2.781 × 2.780	1.391 × 1.391 × 2.780	1 up to 10	4	28	Yes (PSF correction was implemented following the approach from [17])	None	None
TOF + PSF + filter	128 × 128 × 89	256 × 256 × 89	35.6	2.781 × 2.781 × 2.780	1.391 × 1.391 × 2.780	1 up to 10	4	28	Yes (PSF correction was implemented following the approach from [17])	3.5 mm FWHM gaussian in all 3 dimensions	3.5 mm Gaussian transaxial and 3-point axial convolution filter

### Phantom data analysis

Data analysis was performed by placing regions of interest (ROI) using the software package PMOD (version 3.602 & 4.005, PMOD Technologies Ltd., Zurich, Switzerland). A single slice ROI equal to the size of the actual inner sphere diameter was placed in the transaxial plane in which the sphere was most visible (according to the NEMA recommendations) [18]. Furthermore, a spherical VOI with the same diameter as the sphere size was placed on the volumetric sphere image. For the background, 12 ROIs with sizes corresponding to those placed on the spheres were drawn on the same plane where the single slice ROI was placed and at an axial offset of +/− 1 cm and +/− 2 cm, resulting in a total number of 60 background ROIs for each sphere, respectively. Single slice background ROIs were not extended to spherical VOIs.

RCs and %BG variability were determined according to the standardized NEMA NU 2-2007 protocol [18] using the following formulas:

1 $$ {\mathrm{RC}}_{hot}=\frac{\left(\raisebox{1ex}{${C}_{hot\_ sphere}$}\!\left/ \!\raisebox{-1ex}{${C}_{background\_ sphere}$}\right.\right)-1}{\left(\raisebox{1ex}{${A}_{hot}$}\!\left/ \!\raisebox{-1ex}{${A}_{background}$}\right.\right)-1}\times 100\% $$

2 $$ {\mathrm{RC}}_{cold}=\left(1-\frac{C_{cold\_ sphere}}{C_{background\_ sphere}}\right)\times 100\% $$

3 $$ \%\mathrm{BG}\ \mathrm{variability}=\frac{SD_{sphere}}{C_{background\_ sphere}}\times 100\% $$

4 $$ {\mathrm{SD}}_{\mathrm{sphere}}=\sqrt{\sum \limits_{k=1}^K\raisebox{1ex}{${\left({C}_{background\_ sphere,k}-{C}_{background\_ sphere}\right)}^2$}\!\left/ \!\raisebox{-1ex}{$\left(K-1\right)$}\right.},K=60 $$

where *C*_*hot* _ *sphere*_, *C*_*cold* _ *sphere*_, and *C*_*background* _ *sphere*_ correspond to the mean concentration measured in ROIs placed on the images of the hot and cold sphere and on the background for the respective spheres. *A*_*hot*_ and *A*_*background*_ correspond to the true activity concentration measured with a well-counter for the hot spheres and background, respectively.

For the contrast phantom data, the coefficient of variation between voxel values within uniform background regions was used as a measure of voxel noise (%) in the reconstructed images according to the following formula:

5 $$ \mathrm{voxel}\ \mathrm{noise}\ \left(\%\right)=\frac{SD_{background\_ sphere}}{C_{background\_ sphere}}\times 100\% $$

where *SD*_*background* _ *sphere*_ describes the standard deviation measured in ROIs placed on the background for the respective spheres.

The voxel noise (%) was determined for frames with a relatively high number of prompts (~ 135 million), and a low number of prompts (~ 12 million) to mimic count statistics encountered during typical human ^11^C scans. High count data were analyzed based on a single realization while low count data were based on five replicates. Data were reconstructed with up to 10 iterations using the reconstruction parameters as specified above.

### Human scans

Cross-validation studies were performed with six healthy volunteers (age 63.5 ± 15.18 years). The Clinical Research Ethics Board of the University of British Columbia approved the study, and informed written consent was provided by all participating subjects.

Two healthy volunteers were scanned using [^11^C]DTBZ, three with [^18^F]FDG, and one with [^11^C]raclopride on both scanners. The two scans of each subject were performed within 1.5 months except for one [^11^C]DTBZ subject whose time interval between the two scans was 8 months; this was not considered to be a confound as VMAT2 binding shows a negligible age dependence over this time frame [19]. Subjects scanned with [^18^F]FDG fasted for at least 6 h before tracer injection.

On the HRRT, the subjects were positioned using external lasers aligning the gantry with the inferior orbital-external metal line, and custom-fitted thermoplastic masks were applied to minimize head movement. Subject positioning on the SIGNA PET/MR scanner was performed based on an MRI localizer sequence. Intravenous administration of the tracers (see Table 4 for detailed information on injected activity amounts) over 60 s were performed using an infusion pump (Harvard Instruments, Southnatick, MA, USA; one volunteer scanned with [^18^F]FDG was manually injected due to more fragile veins of this subject). Injected activity between scanners was matched for each subject, respectively, with the exception of one scan, where the injected activity was lower for the HRRT scan due to technical reasons (Table 4). List-mode data were acquired for 60 min and histogrammed into 16 frames for [^11^C]DTBZ and [^11^C]raclopride (4 × 60 s, 3 × 120 s, 8 × 300 s, 1 × 600 s) and into 17 frames for [^18^F]FDG (4 × 60 s, 3 × 120 s, 10 × 300 s).

**Table 4:** Injected activity of the respective tracer for each subject at start of the acquisition

Tracer	Subject ID	HRRT [MBq]	SIGNA PET/MR [MBq]
[^11^C]DTBZ	Subject 1	341.3	326.8
	Subject 2	356.1	339.2
[^18^F]FDG	Subject 3	187.3	183.2
	Subject 4	51.3	188.3
	Subject 5	181.4	173.6
[^11^C]raclopride	Subject 6	359.6	378.7

For the HRRT, a transmission scan with a ^137^Cs source was performed before tracer injection. Reconstruction of the HRRT data was performed using OP-OSEM with 16 subsets and 6 iterations (see Table 2). Corrections for decay, dead-time, random, attenuated and scattered coincidences, and detector normalization were applied. Reconstructed images were post-processed with a 2.0-mm FHWM Gaussian filter (standard in-house reconstruction).

For the PET/MR, a zero echo time (ZTE) approach was utilized to correct for attenuation (ZTE MRAC) with sequence parameters: echo time (TE) = 0.016 ms, repetition time (TR) = 399.564 ms, matrix size = 110 × 100 × 116, voxel size = 2.4 × 2.4 × 2.4 mm^3^, flip angle (FA) = 0.8°, number of excitations (NEX) = 4, acquisition time = 42 s [20]. PET/MR data were reconstructed using TOF-OSEM with 28 subsets and 4 iterations, a matrix size of 256 × 256 resulting in a reconstructed voxel size of 1.391 × 1.391 × 2.780 mm^3^ (see Table 3). Based on the results of the phantom study, the post-processing parameters chosen for the human data included the manufacturer supplied 3.5 mm Gaussian transaxial filter as well as a 3-point axial convolution filter and PSF resolution modeling. All manufacturer-provided corrections were applied (dead-time, decay, random, attenuation, and scattered coincidences, normalization).

Reconstructed dynamic PET images were frame-to-frame realigned based on a rigid-body transformation (Statistical Parametric Mapping (SPM), version 12, Wellcome Trust Centre for Neuroimaging, University College London, UK) to correct for potential motion during the scan. Anatomical MR images were co-registered and resampled to the mean PET images using SPM. MR sequence parameters were: 3D brain volume imaging (BRAVO) sequence, TE = 2.984 ms, TR = 7.948 ms, matrix size = 256 × 256 × 162, voxel size = 1 × 1 × 1 mm^3^, FA = 12°, NEX = 1, acquisition time = 6:13 min for healthy volunteer 1-4; magnetization-prepared rapid acquisition gradient echo (MPRAGE) sequence, TE = 3.168 ms, TR = 8.412 ms, MPRAGE TR = 2488 ms, matrix size = 256 × 256 × 164, voxel size = 1 × 1 × 1mm^3^, FA = 8°, NEX = 1, and acquisition time = 7:39 min for healthy volunteer 5-6. Predefined target and reference ROIs based on the Montreal Neurological Institute (MNI) template were eroded and applied to the PET images by using the inverse transformation of the co-registered MR images to the MNI space.

For [^11^C]DTBZ and [^11^C]raclopride scans, non-displaceable binding potentials (BP_ND_) in the caudate (left and right) and for three putamen regions covering the entire length were calculated using the Logan graphical analysis [21] with the occipital cortex and cerebellum, respectively, as reference regions.

[^18^F]FDG binding ratios were determined based on the last 30 min of each dataset using the pons as reference region for the same striatal ROIs used in the evaluations of [^11^C]DTBZ and [^11^C]raclopride and additional regions including the cerebellum, left and right medial front gyrus, medulla, midbrain and occipital cortex to reflect the widespread [^18^F]FDG distribution across the brain.

Regional time activity curves (TACs) were determined for all subjects for two target regions (left caudate and left putamen 1) and the respective reference region (DTBZ: occipital cortex; FDG: pons; raclopride: cerebellum). Radial profiles of a single voxel width were placed on the images averaged over the entire acquisition to determine potential spatial variations between the HRRT and PET/MR data. The activity concentrations along the profiles were normalized to the injected activity.

## Results

### RCs and %BG variability

**Fig. 1:**
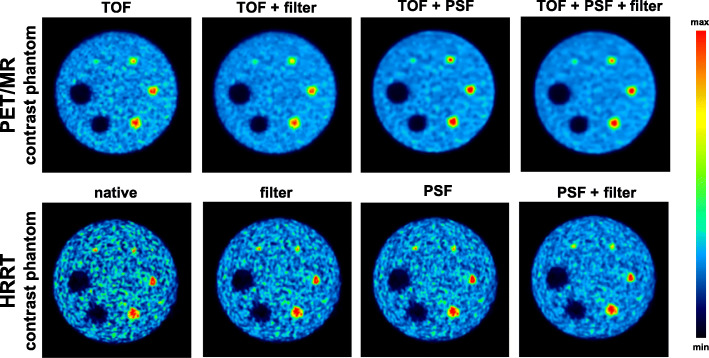
Representative images of the contrast phantom data for different reconstruction parameters for the two systems. Color scales of the images were normalized based on the average background value multiplied by the respective sphere to background ratio

**Fig. 2:**
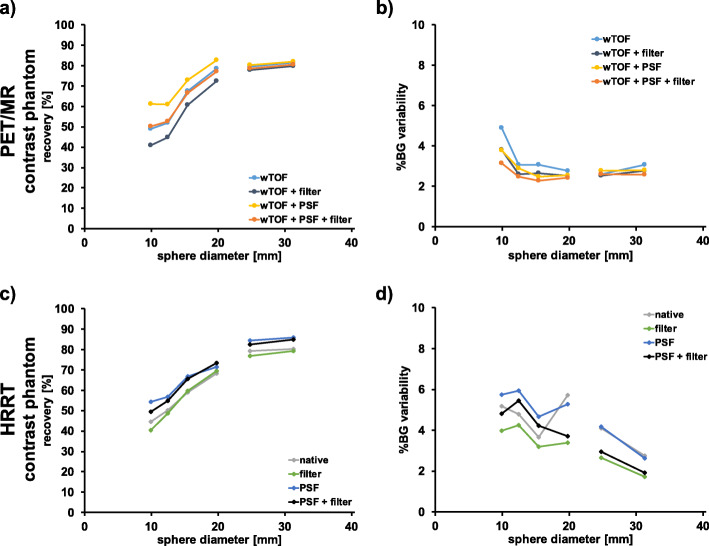
RCs and %BG variability as a function of sphere diameter for the contrast phantom (PET/MR: RCs (**a**), %BG variability (**b**); HRRT: RCs (**c**), %BG variability (**d**)). PET/MR data were reconstructed with TOF, TOF with filter, TOF with PSF, and TOF with PSF and filter. Two iterations and 28 subsets were used, respectively (GE recommendation for phantom data). HRRT data were reconstructed without any filters (native), with a 2-mm Gaussian filter (standard in-house reconstruction for human data) using 6 iterations and 16 subsets, respectively, with PSF, and with PSF correction and 2-mm filter using 10 iterations and 16 subsets, respectively. Note the gap between spheres marks cold vs. hot spheres

Figure 1 shows representative images of the contrast phantom data for different reconstruction parameters. RCs and %BG variability as a function of sphere diameter for different reconstruction settings are depicted in Fig. 2 and listed in Tables 5 and 6. Supplementary Figure 1 and 2 and Supplementary Table 1 depict the corresponding results for the image quality phantom according to NEMA recommendations. As expected, the highest RCs for all hot spheres of the contrast phantom scanned on the PET/MR were obtained from the TOF + PSF reconstruction, while the lowest RCs were obtained by applying post-filtering without PSF correction (Fig. 2a). The cold spheres revealed similar RCs for all 4 evaluated reconstruction settings. %BG variability was highest for TOF only reconstruction and lowest for TOF + PSF + filter reconstruction for most of the spheres (Fig. 2b).

**Table 5:** RCs for the contrast phantom scanned on the PET/MR and HRRT

			Spheres					
			9.9 mm	12.4 mm	15.4 mm	19.8 mm	24.8 mm	31.3 mm
PET/MR	Contrast phantom	wTOF	49.0	51.9	67.7	78.5	79.5	81.4
		wTOF + filter	40.9	44.8	60.8	72.6	77.9	79.9
		wTOF + PSF	61.1	60.9	72.9	82.6	80.4	81.9
		wTOF + PSF + filter	50.2	52.6	66.7	77.2	78.7	80.5
HRRT	Contrast phantom	Native	44.5	50.1	59.1	68.4	79.3	80.4
		Filter	40.4	48.7	59.6	69.4	76.8	79.3
		PSF	54.4	56.9	66.9	71.6	84.5	85.9
		PSF + filter	49.5	54.9	65.6	73.4	82.5	85.0

**Table 6:** %BG variability for the contrast phantom scanned on the PET/MR and HRRT

			Spheres					
			9.9 mm	12.4 mm	15.4 mm	19.8 mm	24.8 mm	31.3 mm
PET/MR	Contrast phantom	wTOF	4.7	3.0	3.0	2.7	2.5	2.9
		wTOF + filter	3.6	2.5	2.6	2.4	2.4	2.6
		wTOF + PSF	3.6	2.8	2.4	2.5	2.7	2.7
		wTOF + PSF + filter	3.0	2.4	2.2	2.3	2.5	2.5
HRRT	Contrast phantom	Native	5.0	4.6	3.5	5.5	3.9	2.7
		Filter	3.8	4.1	3.1	3.2	2.6	1.6
		PSF	5.5	5.7	4.5	5.1	4.0	2.5
		PSF + filter	4.6	5.2	4.1	3.6	2.8	1.8

For the contrast phantom scanned on the HRRT (Fig. 2c), similar RCs were obtained from the PSF reconstruction with and without filter for all spheres except for the smallest one (PSF: 54.4%; PSF + filter: 49.5%). Lowest RCs for the four hot spheres were observed from the native and the 2-mm Gaussian post filter reconstruction. As expected, the relatively narrow post filter had the biggest impact on the RC of the smallest sphere. The filtered reconstruction yielded lowest %BG variability values for all spheres (Fig. 2d).

In direct comparison of the contrast phantom data, both the PET/MR TOF only and TOF + PSF + filter reconstructions revealed similar RCs for the hot spheres compared to both the HRRT PSF and PSF + filter reconstructions (Fig. 2 and Tables 5 and 6), except for the smallest sphere for the HRRT PSF reconstruction (deviations ~ 10% between scanners).  The PET/MR TOF + filter reconstruction revealed similar RC values compared to the HRRT filter only reconstruction. 

RCs of the PET/MR decreased for all sphere sizes when the TOF information was not used (woTOF, Supplementary Figure 3 and Supplementary Table 2).

RCs of all hot spheres decreased for the spherical VOI analysis in comparison to the single slice ROI analysis (except for the largest hot sphere for the HRRT PSF reconstruction, Supplementary Figure 4).

### RC and voxel-level noise (%)

**Fig. 3:**
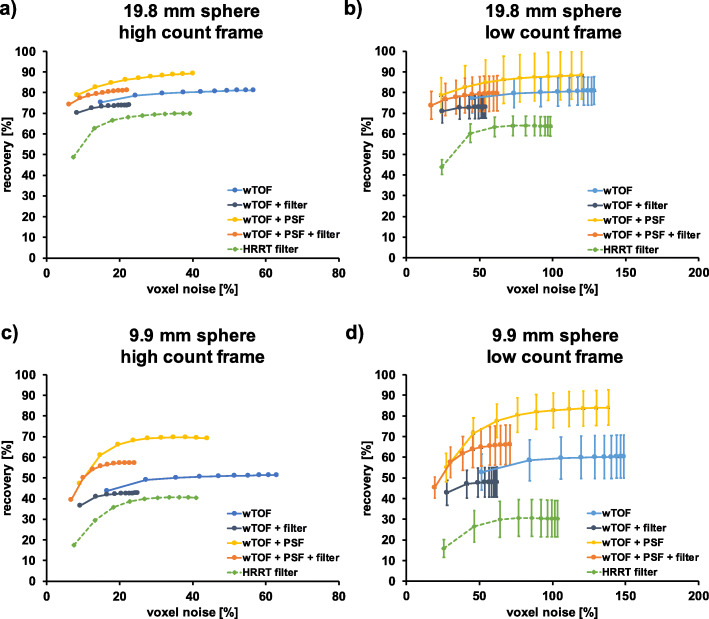
PET/MR RCs (%) as a function of voxel noise (%) for two different sphere sizes and for a high and low count frame for the contrast phantom (19.8 mm sphere: high count frame (**a**), low count frame (**b**); 9.9 mm sphere: high count frame (**c**), low count frame (**d**)). The high count frame corresponds to ~ 135 million prompts within the frame, the low count frame to ~ 12 million prompts. Note the difference in *x*-axis limits. PET/MR data were reconstructed with TOF, TOF with filter, TOF with PSF, and TOF with PSF and filter using 28 subsets, respectively. High count data were analyzed based on a single realization while low count data were based on five replicates. Each point represents an OSEM iteration, and the number of iterations increases from left to right. The dotted line represents the HRRT standard in-house reconstruction (2-mm Gaussian filter) used for human data reconstruction

**Fig. 4:**
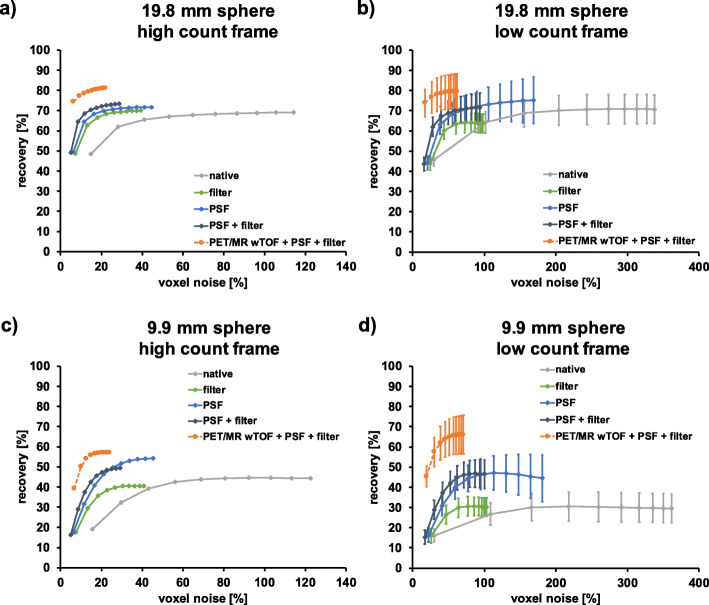
HRRT RCs (%) as a function of voxel noise (%) for two different sphere sizes and for a high and low count frame for the contrast phantom (19.8 mm sphere: high count frame (**a**), low count frame (**b**); 9.9 mm sphere: high count frame (**c**), low count frame (**d**)). The high count frame corresponds to ~ 135 million prompts within the frame, the low count frame to ~ 12 million prompts. Note the difference in *x*-axis limits. HRRT data were reconstructed without any filters (native), with a 2-mm Gaussian filter (standard in-house reconstruction for human data), with PSF, and with PSF correction and 2-mm filter using 16 subsets, respectively. High count data were analyzed based on a single realization while low count data were based on five replicates. Each point represents an OSEM iteration, and the number of iterations increases from left to right. The dotted line represents the PET/MR reconstruction (wTOF, PSF, 3.5-mm Gaussian filter) used for human data reconstruction

RCs versus voxel noise (%) comparison between various reconstruction parameters, two different sphere sizes, and count statistics for the contrast phantom scanned on the PET/MR and the HRRT is illustrated in Figs. 3 and 4, respectively. A systematic increase in voxel noise (%) was observed for the low count frame in comparison to the high count frame. The highest noise levels for the PET/MR were obtained for the TOF only reconstruction, whereas the lowest noise levels were obtained when using TOF with filter and TOF with PSF and filtering. The HRRT native reconstruction revealed the highest voxel noise (%), while the lowest voxel noise (%) was determined for both filtered reconstructions.

**Fig. 5:**
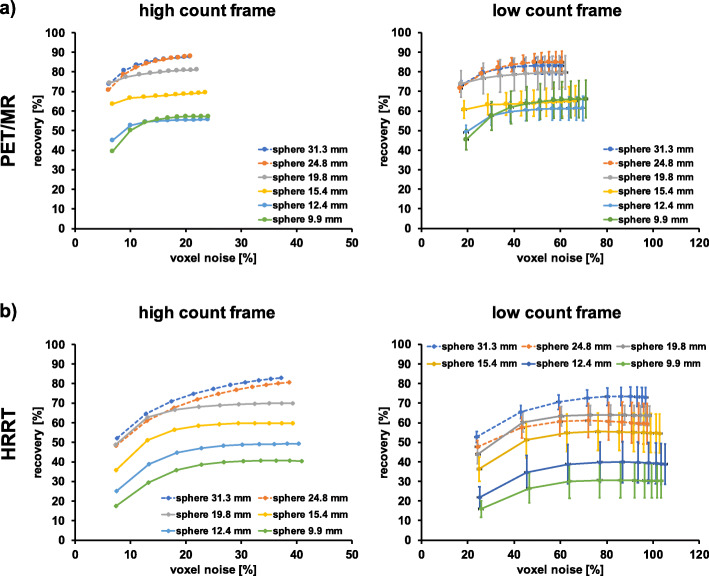
RCs (%) as a function of voxel noise (%) for all sphere sizes for a high and low count frame for the contrast phantom (PET/MR (**a**), HRRT (**b**)). The high count frame corresponds to ~ 135 million prompts within the frame, the low count frame to ~ 12 million prompts. Note the difference in *x*-axis limits. Each point represents an OSEM iteration, and the number of iterations increases from left to right. PET/MR data were reconstructed with TOF + PSF + filter; HRRT data were reconstructed with filter only (standard in-house reconstruction for human data). High count data were analyzed based on a single realization while low count data were based on five replicates. Solid lines represent the hot spheres, dotted lines the cold spheres

Figure 5 shows the RCs (%) versus voxel noise (%) for all sphere sizes based on the reconstruction parameters used for the human data. Increased voxel noise (%) for the HRRT compared to the PET/MR was found for the high and low count frame. For the PET/MR, the trajectory of RCs versus voxel noise (%) reached a relatively stable value from iteration 4 on for most of the hot spheres; reconstructing with more iterations increased noise but not RCs significantly.

Based on the phantom results, the best trade-off in terms of RCs, %BG variability, and voxel noise (%) for the PET/MR was achieved with the reconstruction setting of TOF + PSF + filter and 4 iterations (lowest %BG variability, voxel noise in % vs RCs for almost all spheres converged along with high RCs due to PSF correction), which was consequently used for the reconstruction of all human data.

### Human scans

**Fig. 6:**
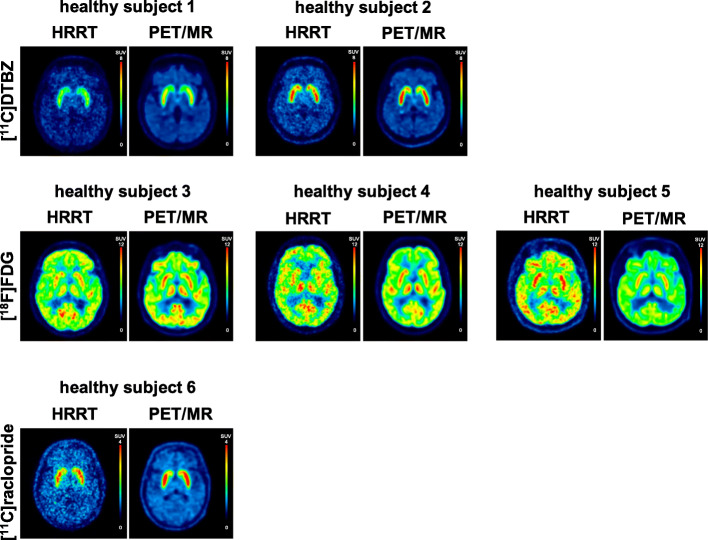
Representative images of the mean [^11^C]DTBZ, [^18^F]FDG, and [^11^C]raclopride tracer distribution for each subject. Color scales of the images were adjusted based on SUV values for each tracer separately with a common maximum between scanners. PET/MR data were reconstructed with TOF with PSF and filter using 4 iterations and 28 subsets; HRRT data were reconstructed using 6 iterations and 16 subsets and post-processed with a 2.0 mm FHWM Gaussian filter (standard in-house reconstruction). Note: Subject 4 was injected with a lower amount of [^18^F]FDG activity for the HRRT scan compared to the other scans; hence, noise characteristics differ accordingly

**Fig. 7:**
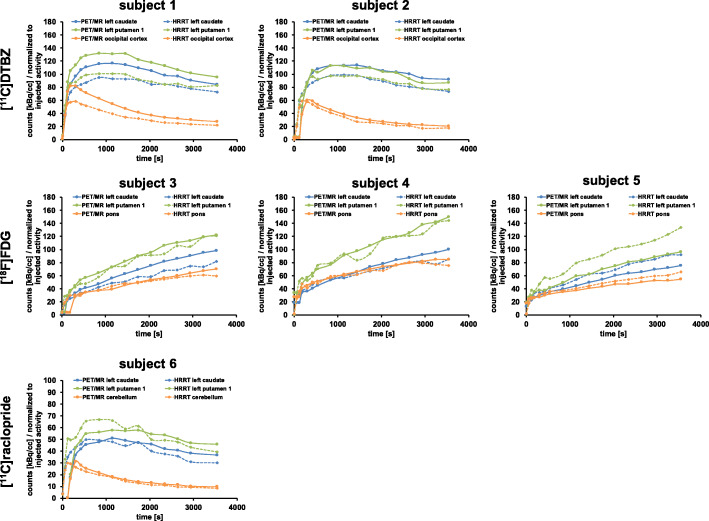
Regional time activity curves for each subject for two target regions (left caudate and left putamen 1) and the reference region ([^11^C]DTBZ: occipital cortex, [^18^F]FDG: pons, [^11^C]raclopride: cerebellum) for all subjects. Note that the offset in time activity curves between the PET/MR and HRRT is due to different acquisition start triggering mechanisms (HRRT: manual acquisition start; PET/MR: acquisition start based on counts). Solid lines represent the PET/MR data, dotted lines the HRRT data

Figure 6 depicts representative images of the mean [^11^C]DTBZ, [^18^F]FDG, and [^11^C]raclopride tracer distribution for each tracer. Figure 7 illustrates regional time-activity curves for each subject. A voxel-wise correlation of the HRRT to PET/MR activity concentration for each subject can be found in Supplementary Figure 5.

### Line profiles

**Fig. 8:**
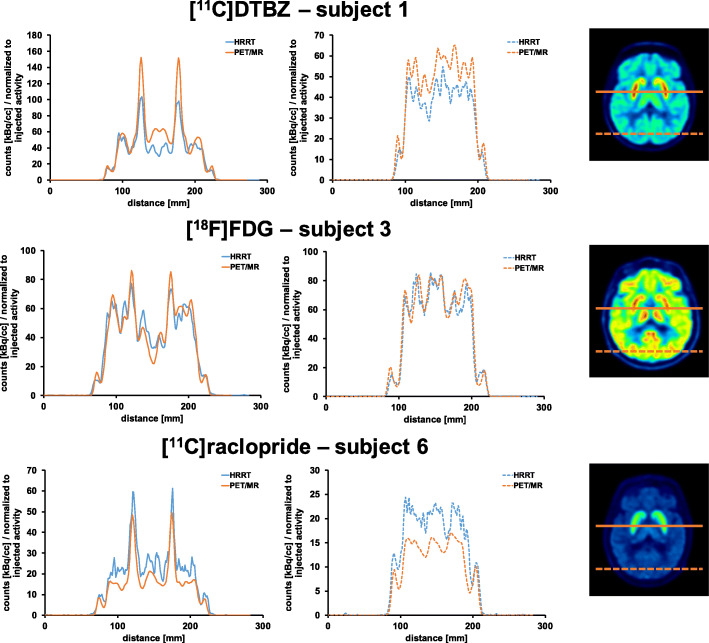
Line profiles ([^11^C]DTBZ, [^18^F]FDG, [^11^C]raclopride) of mean PET images. Profiles were normalized to the injected activity of each subject. Position of line profiles along the FOV (solid lines: typical target regions; dotted lines: typical reference regions) are illustrated on a representative slice of the mean [^11^C]DTBZ, [^18^F]FDG and [^11^C]raclopride image

Line profiles show excellent spatial agreement between the images obtained with the two scanners (Fig. 8). [^11^C]DTBZ and [^11^C]raclopride profiles reveal partial magnitude differences, while [^18^F]FDG profiles were almost identical.

### [^11^C]DTBZ

**Fig. 9:**
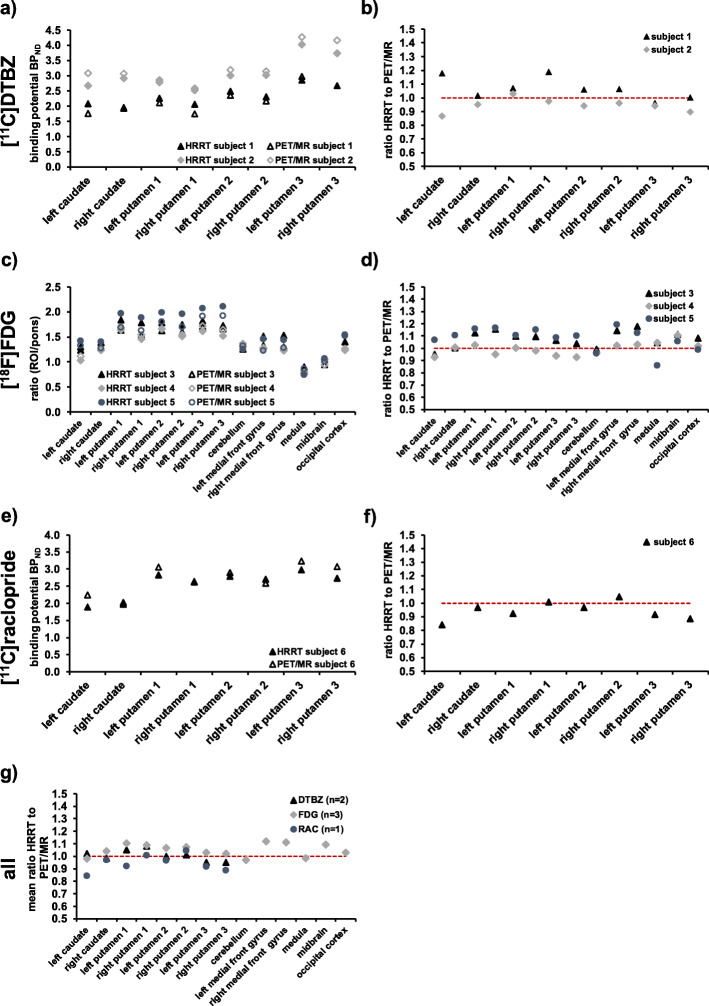
BP_ND_ of [^11^C]DTBZ (**a**) and [^11^C]raclopride (**e**) scans and ratios to reference region for [^18^F]FDG (**c**) scans. Calculated ratios of HRRT to PET/MR scans are depicted for [^11^C]DTBZ (**b**), [^18^F]FDG (**d**), and [^11^C]raclopride (**f**). Mean ratios of HRRT to PET/MR values for each tracer are depicted in (**g**). Dotted red line represents the ratio of 1

The estimated BP_ND_ values obtained on the two scanners were found to be in good agreement and deviations were below 10% except for the left caudate for both subjects, the right anterior putamen for subject 1 and the right posterior putamen for subject 2 (Fig. 9a and b).

### [^18^F]FDG

Overall, the ratios between different regions and pons were comparable between scanners (Fig. 9c and d). Deviations larger than 10% were detected for the anterior left and right putamen and left and right medial front gyrus for subject 3 and subject 5, respectively, and for the midbrain for subject 3 and the right putamen 2, as well as the medulla for subject 5.

### [^11^C]raclopride

BP_ND_ values revealed deviations below 10% except for the left caudate and the right posterior putamen (Fig. 9e and f).

Figure 9g displays mean HRRT to PET/MR parameters of all subjects for each tracer, respectively. Mean [^11^C]DTBZ ratios revealed deviations below 10% for all investigated regions. The same was detected for the mean [^18^F]FDG ratios except for the left anterior putamen (10.4%) and left and right medial front gyrus (11.9% and 11.1%, respectively). [^11^C]raclopride ratios were determined based on a single subject.

## Discussion

Cross-validation studies between the HRRT and the SIGNA PET/MR system were performed to assess the comparability with a specific focus on brain imaging.

For phantom studies, different PET/MR reconstruction parameters were tested to evaluate those most suitable for in vivo studies, though the scope of the study was not to necessarily perform a full optimization of the reconstruction and post-processing parameters, but rather to evaluate parameters that can be routinely used for human data acquisition.

As expected, the highest RCs were observed from PSF reconstructions for both PET/MR and HRRT data (Fig. 2) as has been reported by multiple studies for clinical PET systems [8, 16, 22–25]. However, since resolution modeling with PSF can cause edge artifacts (Gibbs artifacts), PSF reconstruction is not necessarily always the most quantitatively accurate method and it needs to be carefully evaluated [16, 22, 26, 27]. %BG variability was lowest when applying post-filtering for both scanners and this concurrently resulted in a decrease in RCs (Fig. 2).

Reconstruction with TOF only and with TOF, PSF correction and filter, revealed similar RCs for the PET/MR. This is likely due to fact that the improvement in RCs due the resolution modeling is reversed due to the post-processing filter with the chosen width. The net result was noise reduction without altering RCs. Applying a different filter width would likely yield differences in RCs between TOF only and TOF, PSF correction + filter.

The HRRT %BG variability was slightly higher compared to the PET/MR for most of the spheres (Fig. 2b and d). Voxel noise (%) of HRRT images was significantly higher (Fig. 5). As the HRRT uses smaller detector crystals compared to the PET/MR and the reconstructed voxel size was also smaller, higher noise levels both on a regional and voxel-level are expected due to the lower measured counts per detector pair.

Determined voxel noise (%) for the HRRT (Fig. 5) is comparable to already published results [28] within the limits of small differences in the phantom used and methodological approaches.

While the HRRT clearly outperforms the PET/MR in terms of intrinsic spatial resolution and resolution uniformity, the PET/MR TOF capability leads to significantly improved signal-to-noise ratios [29, 30]. It has been reported, that TOF reconstruction results in higher RCs at matched noise levels with faster convergence when comparing to woTOF data [31–33]. This is in line with our results (Fig. 5). Furthermore, when comparing RCs reconstructed with and without TOF, all spheres (Supplementary Figure 3 and Supplementary Table 2), revealed a decrease in RCs when no TOF information was used for reconstruction, as previously reported [30, 34, 35]. The cold spheres exhibited a huge decrease in RCs when TOF information was not utilized for reconstruction, which is in line with published results [8, 36]. Furthermore, comparing the woTOF PET/MR data to the HRRT data revealed lower RCs for all PET/MR reconstructions for the two smallest hot spheres due to the lower spatial resolution of the PET/MR system (Supplementary Figure 3 and Supplementary Table 2). Hence, the use of TOF information for reconstruction contributes to partially off-set the intrinsically higher spatial resolution of the HRRT system.

RCs were determined according to the NEMA protocol and are based on a single slice ROI; the single slice ROI shall be positioned on the image slice with the highest sphere activity concentration [18]. However, this might be prone to inaccuracies, as the center of the sphere might be between two adjacent slices (axial slice thickness PET/MR: 2.78 mm; HRRT: 1.22 mm). A VOI-based analysis method covering the entire sphere was proposed as a more robust analysis method [37]. Determining the recovery with a VOI-based method revealed lower RCs for all hot spheres for the PET/MR and HRRT (except for the largest hot sphere PSF reconstruction), though changes in RCs due to the analysis method were larger for the PET/MR (Supplementary Figure 4). Our results are in line with results from the literature [37], demonstrating that the analysis method clearly has a significant impact on the determination of the RCs.

Within the limitations discussed above (different reconstructed voxel sizes between scanners, spatial resolution of the scanners and reconstruction parameters (TOF vs. woTOF, etc.)), a direct comparison of the phantom data between the HRRT and PET/MR revealed comparable RCs, %BG variability, though voxel noise (%) differed significantly. The best trade-off (lowest %BG variability, voxel noise in % vs RCs for almost all spheres was converged along with high RCs due to PSF correction) between RCs, %BG variability and voxel noise (%) for the PET/MR was found with PSF correction and filtering. Hence, this reconstruction paradigm was used for reconstruction of all subject scans and compared to results from our own standard HRRT reconstruction setting used for patient scans (2 mm Gaussian filtering).

TACs of the HRRT subject scans revealed higher noise than the PET/MR TACs, due to the smaller crystals, as well as smaller reconstructed voxel sizes (Fig. 7), consistent with the phantom data. No systematic differences between line profiles were detected (Fig. 8), indicating that differences in profiles are likely due to scan-to-scan or biological variations. This also indicates that the quantitative data correction algorithms implemented on each scanner do not contribute to significant acquisition and scanner specific biases.

The variation of the [^11^C]DTBZ BP_ND_ was found to be within 10% (Fig. 9g). Although the time interval between both scans for subject 1 was 8 months, no influence of the extended time interval was detected, which is in line with literature reporting that VMAT2 binding shows a negligible age dependence over this time frame [19].

Similar differences as for [^11^C]DTBZ were found for [^18^F]FDG ratios for most of the investigated regions, which were spanning a wider fraction of the brain. The largest deviation was detected for the medial front gyrus possibly due to the differences in spatial resolution uniformity across the FOV [8, 12]. Considering also the fact that calculated deviations are based on 3 subjects only, we can conclude that both systems revealed very similar [^18^F]FDG ratios.

In general, a trend towards larger differences between both systems for the posterior putamen was observed. This region is affected most by the partial volume effect, as it is the smallest region investigated in this study [38]. As the HRRT has a higher spatial resolution, differences in BP_ND_, as well as ratios, could be expected to be the largest for this region.

Comparison of the acquired healthy subject data might be biased due to different attenuation correction approaches used for the HRRT and PET/MR data. HRRT subject data were corrected for attenuation using a ^137^Cs transmission scan approach, whereas the PET/MR subject data were corrected based on a ZTE MRI scan of the respective scan. Transmission scans are in general more sensitive to noise, as the signal to noise ratio solely depends on the acquisition duration of the transmission scan and the activity of the used transmission source. Furthermore, Sousa et al. determined higher linear attenuation coefficients in brain imaging for ZTE attenuation maps compared to ^68^Ge attenuation maps. For the cerebellum and posterior cortical regions, a higher correlation and improved precision in standard uptake values were detected when ZTE attenuation maps were used compared to ^68^Ge transmission scans [39]. Multiple studies have shown that this is due to an improved estimation of the temporal bone in ZTE maps [20, 39, 40]. However, based on the evaluated line profiles as shown in Fig. 8, no significant and systematic impact due to different attenuation correction approaches was determined.

Smaller voxel sizes were chosen for the reconstruction of PET/MR subjects’ scans compared to phantom data to enable the VOI position based on a finer pixel grid. Voxel size was determined to have no impact on the RCs (deviation of RCs between 128 and 256 matrix size was below 1.6%).

Several studies have reported that variability (instrumentation and biological based) in test-retest scans of multiple applications is approximately 10% or more [41–44]. Given that the number of subjects in our study was relatively low, coupled with the outcomes of the phantom studies, it is reasonable to expect that the estimate of variability between same-subject scans performed on the two scanners may even decrease when increasing the number of subjects.

It is of interest to note that our results most likely are applicable to a comparison between the HRRT and a GE PET/CT system, as both the GE PET/MR and PET/CT systems are based on a similar detector design and reconstruction algorithms [45]. Especially the 5-ring configuration of the PET/CT system with the same axial FOV as the PET/MR will most likely demonstrate similar in vivo image quality parameters [45].

## Conclusions

This work demonstrates that the whole-body hybrid SIGNA PET/MR system is well suited for high-resolution human brain imaging and that the scanner performance in a clinical setting is quite comparable to that of the HRRT system, which is still arguably the human scanner with the highest intrinsic resolution. The HRRT outperforms the PET/MR in terms of spatial resolution, but exhibits a higher voxel noise (%) due to its smaller crystals and smaller reconstructed voxel sizes and lack of TOF capability; the PET/MR TOF capability indeed contributed to off-set to some degree the higher spatial resolution of the HRRT in terms of overall image quality.

BP_ND_ of [^11^C]DTBZ subjects, as well as ratios of [^18^F]FDG scans, revealed a variability between both scans of less than 12%, which is in the range of typical test-retest variability. From a clinical point of view and based on our results, we expect the two scanners to provide similar results in regard to brain imaging. The main difference between the two scanners is the attenuation correction approach with the HRRT scanner using a transmission source scan, whereas the PET/MR is based on a ZTE MRI scan. Based on our results, this was not a confound in comparing the two scanners. A significant clear advantage of the PET/MR however remains the fact that the simultaneous imaging approach of PET and MR enables the acquisition of multiple parameters with the brain in the same physiological state, in addition to providing an anatomical reference required for several image analysis approaches.

## Supplementary Information

**Additional file 1: Supplementary Figure 1**: Representative images of the image quality phantom data for different reconstruction parameters. Color scales of the images were normalized based on the average background value multiplied by the respective sphere to background ratio. **Supplementary Figure 2**: RCs (a) and %BG variability (b) as a function of sphere diameter for the image quality phantom. PET/MR data were reconstructed with TOF, TOF with filter, TOF with PSF, and TOF with PSF and filter. 2 iterations and 28 subsets were used, respectively (GE recommendation for phantom data). Note the gap between spheres marks cold vs. hot spheres. **Supplementary Figure 3**: RCs (%) of the PET/MR (a) as a function of sphere diameter for the contrast phantom with (solid lines) and without TOF (dotted lines). Comparison of RCs (b) without TOF of the PET/MR (solid lines) to the HRRT (dotted lines). Note the gap between spheres marks cold vs. hot spheres. **Supplementary Figure 4**: RCs (%) (PET/MR (a), HRRT (b)) versus sphere diameter for the contrast phantom. Data were analyzed with a single slice ROI (solid lines), the standard NEMA analysis method, and with a spherical VOI matching the physical sphere diameter (dotted lines). Note the gap between spheres marks cold vs. hot spheres. **Supplementary Figure 5**: Voxel-wise correlation of the HRRT to PET/MR activity concentration for each subject ([^11^C]DTBZ, [^18^F]FDG, [^11^C]raclopride). The black line indicates the identity line, the red dotted line displays the linear regression of the values with the corresponding R^2^. **Supplementary Table 1**: RCs (a) and %BG variability (b) for the image quality phantom scanned on the PET/MR. **Supplementary Table 2**: RCs for the contrast phantom scanned on the PET/MR and HRRT. PET/MR data were reconstructed with and without TOF information.

## Data Availability

The phantom data are available upon request. The human data are not publicly available due to subject confidentiality.

## Abbreviations

[^11^C]DTBZ: [^11^C]dihydrotetrabenazine
[^18^F]FDG: [^18^F]-fluorodeoxyglucose
%BG: Percent background variability
BRAVO: Brain volume imaging
BP_ND_: Non-displaceable binding potentials
cFOV: Center of the field of view
CT: Computed tomography
DOI: Depth-of-interaction
FA: Flip angle
FBP: Filtered backprojection
FWHM: Full width at half maximum
HRRT: High-resolution research tomograph
ID: Inner diameter
LSO: Lutetium oxyorthosilicate
LYSO: Lutetium-yttrium oxyorthosilicate
MNI: Montreal Neurological Institute
MPRAGE: Magnetization-prepared rapid acquisition gradient echo
MRI: Magnetic resonance imaging
NEMA: National Electrical Manufacturers Association
NEX: Number of excitations
OP-OSEM: Ordinary Poisson ordered subset expectation maximization
PET: Positron emission tomography
PMT: Photomultiplier tube
PSF: Point spread function
RC: Recovery coefficient
ROI: Regions of interest
SiPM: Silicon photomultiplier
SPM: Statistical Parametric Mapping
TE: Echo time
TOF: Time-of-flight
TR: Repetition time
VMAT2: Vesicular monoamine transporter 2
ZTE: Zero echo time

## References

[1] Cherry SR, Jones T, Karp JS, Qi J, Moses WW, Badawi RD (2018). Total-body PET: maximizing sensitivity to create new opportunities for clinical research and patient care. J Nucl Med..

[2] Vaquero JJ, Kinahan P (2015). Positron emission tomography: current challenges and opportunities for technological advances in clinical and preclinical imaging systems. Annu Rev Biomed Eng..

[3] Keng FY (2004). Clinical applications of positron emission tomography in cardiology: a review. Ann Acad Med Singapore..

[4] Politis M, Piccini P (2012). Positron emission tomography imaging in neurological disorders. J Neurol..

[5] Bomanji JB, Costa DC, Ell PJ (2001). Clinical role of positron emission tomography in oncology. Lancet Oncol..

[6] Anton-Rodriguez JM, Julyan P, Djoukhadar I, Russell D, Evans DG, Jackson A (2019). Comparison of a standard resolution PET-CT scanner with an HRRT brain scanner for imaging small tumors within the head. IEEE Transactions on Radiation and Plasma Medical Sciences..

[7] Sossi V, Jong HWAMd, Barker WC, Bloomfield P, Burbar Z, Camborde M, et al., editors. The second generation HRRT - a multi-centre scanner performance investigation. IEEE Nuclear Science Symposium Conference Record, 2005; 2005 23-29 Oct. 2005.

[8] Grant AM, Deller TW, Khalighi MM, Maramraju SH, Delso G, Levin CS (2016). NEMA NU 2-2012 performance studies for the SiPM-based ToF-PET component of the GE SIGNA PET/MR system. Med Phys..

[9] Deller TW, Khalighi MM, Jansen FP, Glover GH (2018). PET imaging stability measurements during simultaneous pulsing of aggressive MR sequences on the SIGNA PET/MR system. J Nucl Med..

[10] Barthel H, Schroeter ML, Hoffmann KT, Sabri O (2015). PET/MR in dementia and other neurodegenerative diseases. Semin Nucl Med..

[11] Sander CY, Hansen HD, Wey HY (2020). Advances in simultaneous PET/MR for imaging neuroreceptor function. J Cereb Blood Flow Metab..

[12] de Jong HW, van Velden FH, Kloet RW, Buijs FL, Boellaard R, Lammertsma AA (2007). Performance evaluation of the ECAT HRRT: an LSO-LYSO double layer high resolution, high sensitivity scanner. Phys Med Biol..

[13] Levin CS, Maramraju SH, Khalighi MM, Deller TW, Delso G, Jansen F (2016). Design features and mutual compatibility studies of the time-of-flight PET capable GE SIGNA PET/MR system. IEEE Trans Med Imaging..

[14] Comtat C, Bataille F, Michel C, Jones J, Sibomana M, Janeiro L, et al. OSEM-3D reconstruction strategies for the ECAT HRRT2004. 3492-6 Vol. 6 p.

[15] Comtat C, Sureau F, Sibomana M, Hong I, Sjoholm N, Trébossen R (2008). Image based resolution modeling for the HRRT OSEM reconstructions software.

[16] Blinder SA, Dinelle K, Sossi V (2012). Scanning rats on the high resolution research tomograph (HRRT): a comparison study with a dedicated micro-PET. Med Phys..

[17] Alessio AM, Stearns CW, Tong S, Ross SG, Kohlmyer S, Ganin A (2010). Application and evaluation of a measured spatially variant system model for PET image reconstruction. IEEE Trans Med Imaging..

[18] National Electrical Manufacturers Association (2007). NEMA NU-2 Standards Publication NU-2-2007: Performance measurements of positron emission tomography.

[19] Frey KA, Koeppe RA, Kilbourn MR, Vander Borght TM, Albin RL, Gilman S (1996). Presynaptic monoaminergic vesicles in Parkinson’s disease and normal aging. Ann Neurol..

[20] Yang J, Wiesinger F, Kaushik S, Shanbhag D, Hope TA, Larson PEZ (2017). Evaluation of sinus/edge-corrected zero-echo-time-based attenuation correction in brain PET/MRI. J Nucl Med..

[21] Logan J, Fowler JS, Volkow ND, Wolf AP, Dewey SL, Schlyer DJ (1990). Graphical analysis of reversible radioligand binding from time-activity measurements applied to [N-11C-methyl]-(-)-cocaine PET studies in human subjects. J Cereb Blood Flow Metab..

[22] Oen SK, Aasheim LB, Eikenes L, Karlberg AM (2019). Image quality and detectability in Siemens Biograph PET/MRI and PET/CT systems-a phantom study. EJNMMI Phys..

[23] Aklan B, Oehmigen M, Beiderwellen K, Ruhlmann M, Paulus DH, Jakoby BW (2016). Impact of point-spread function modeling on PET image quality in integrated PET/MR hybrid imaging. J Nucl Med..

[24] Caribe P, Koole M, D'Asseler Y, Deller TW, Van Laere K, Vandenberghe S (2019). NEMA NU 2-2007 performance characteristics of GE Signa integrated PET/MR for different PET isotopes. EJNMMI Phys..

[25] Mansor S, Pfaehler E, Heijtel D, Lodge MA, Boellaard R, Yaqub M (2017). Impact of PET/CT system, reconstruction protocol, data analysis method, and repositioning on PET/CT precision: an experimental evaluation using an oncology and brain phantom. Med Phys..

[26] Tsutsui Y, Awamoto S, Himuro K, Umezu Y, Baba S, Sasaki M (2017). Edge artifacts in point spread function-based PET reconstruction in relation to object size and reconstruction parameters. Asia Ocean J Nucl Med Biol..

[27] Zeng GL (2011). Gibbs artifact reduction by nonnegativity constraint. J Nucl Med Technol..

[28] Cheng JK, Matthews J, Sossi V, Anton-Rodriguez J, Salomon A, Boellaard R (2017). Incorporating HYPR de-noising within iterative PET reconstruction (HYPR-OSEM). Phys Med Biol..

[29] Surti S (2015). Update on time-of-flight PET imaging. J Nucl Med..

[30] Vandenberghe S, Mikhaylova E, D’Hoe E, Mollet P, Karp JS (2016). Recent developments in time-of-flight PET. EJNMMI Phys..

[31] Karp JS, Surti S, Daube-Witherspoon ME, Muehllehner G (2008). Benefit of time-of-flight in PET: experimental and clinical results. J Nucl Med..

[32] Conti M (2009). State of the art and challenges of time-of-flight PET. Phys Med..

[33] Conti M, Bendriem BJC, Imaging T (2019). The new opportunities for high time resolution clinical TOF PET. J Clin Transl Imaging..

[34] Lois C, Jakoby BW, Long MJ, Hubner KF, Barker DW, Casey ME (2010). An assessment of the impact of incorporating time-of-flight information into clinical PET/CT imaging. J Nucl Med..

[35] El Fakhri G, Surti S, Trott CM, Scheuermann J, Karp JS (2011). Improvement in lesion detection with whole-body oncologic time-of-flight PET. J Nucl Med..

[36] Soderlund AT, Chaal J, Tjio G, Totman JJ, Conti M, Townsend DW (2015). Beyond 18F-FDG: characterization of PET/CT and PET/MR scanners for a comprehensive set of positron emitters of growing application--18F, 11C, 89Zr, 124I, 68Ga, and 90Y. J Nucl Med..

[37] Ghahremani A, Bharkhada D, Conti M (2019). Novel volume based approach to estimate contrast recovery for NEMA image quality phantom.

[38] Soret M, Bacharach SL, Buvat I (2007). Partial-volume effect in PET tumor imaging. J Nucl Med..

[39] Sousa JM, Appel L, Engstrom M, Papadimitriou S, Nyholm D, Larsson EM (2018). Evaluation of zero-echo-time attenuation correction for integrated PET/MR brain imaging-comparison to head atlas and (68)Ge-transmission-based attenuation correction. EJNMMI Phys..

[40] Sekine T, Ter Voert EE, Warnock G, Buck A, Huellner M, Veit-Haibach P (2016). Clinical evaluation of zero-echo-time attenuation correction for brain 18F-FDG PET/MRI: comparison with atlas attenuation correction. J Nucl Med..

[41] Doot RK, Scheuermann JS, Christian PE, Karp JS, Kinahan PE (2010). Instrumentation factors affecting variance and bias of quantifying tracer uptake with PET/CT. Med Phys..

[42] Chow TW, Mamo DC, Uchida H, Graff-Guerrero A, Houle S, Smith GS (2009). Test-retest variability of high resolution positron emission tomography (PET) imaging of cortical serotonin (5HT2A) receptors in older, healthy adults. BMC Med Imaging..

[43] Lodge MA (2017). Repeatability of SUV in oncologic (18)F-FDG PET. J Nucl Med..

[44] Fahey FH, Kinahan PE, Doot RK, Kocak M, Thurston H, Poussaint TY (2010). Variability in PET quantitation within a multicenter consortium. Med Phys..

[45] Pan T, Einstein SA, Kappadath SC, Grogg KS, Lois Gomez C, Alessio AM (2019). Performance evaluation of the 5-Ring GE Discovery MI PET/CT system using the national electrical manufacturers association NU 2-2012 Standard. Med Phys..

