# Supplementary material for: Cross-validation study between the HRRT and the PET component of the SIGNA PET/MRI system with focus on neuroimaging
Source: EJNMMI Phys. 2021 Feb 26;8:20. doi: 10.1186/s40658-020-00349-0 (PMC7910400; doi:10.1186/s40658-020-00349-0)
Supplement: Supplementary file 1 — Additional file 1: Supplementary Figure 1: Representative images of the image quality phantom data for different reconstruction parameters. Color scales of the images were normalized based on the average background value multiplied by the respective sphere to background ratio. Supplementary Figure 2: RCs (a) and %BG variability (b) as a function of sphere diameter for the image quality phantom. PET/MR data were reconstructed with TOF, TOF with filter, TOF with PSF, and TOF with PSF and filter. 2 iterations and 28 subsets were used, respectively (GE recommendation for phantom data). Note the gap between spheres marks cold vs. hot spheres. Supplementary Figure 3: RCs (%) of the PET/MR (a) as a function of sphere diameter for the contrast phantom with (solid lines) and without TOF (dotted lines). Comparison of RCs (b) without TOF of the PET/MR (solid lines) to the HRRT (dotted lines). Note the gap between spheres marks cold vs. hot spheres. Supplementary Figure 4: RCs (%) (PET/MR (a), HRRT (b)) versus sphere diameter for the contrast phantom. Data were analyzed with a single slice ROI (solid lines), the standard NEMA analysis method, and with a spherical VOI matching the physical sphere diameter (dotted lines). Note the gap between spheres marks cold vs. hot spheres. Supplementary Figure 5: Voxel-wise correlation of the HRRT to PET/MR activity concentration for each subject ([11C]DTBZ, [18F]FDG, [11C]raclopride). The black line indicates the identity line, the red dotted line displays the linear regression of the values with the corresponding R2. Supplementary Table 1: RCs (a) and %BG variability (b) for the image quality phantom scanned on the PET/MR. Supplementary Table 2: RCs for the contrast phantom scanned on the PET/MR and HRRT. PET/MR data were reconstructed with and without TOF information. [file 40658_2020_349_MOESM1_ESM.pdf]

Supplementary Information

Cross-validation study between the HRRT and the PET component of the SIGNA PET/MRI system with focus on neuroimaging

European Journal of Nuclear Medicine and Molecular Imaging Physics

Julia G. Mannheim[1,2,3*§], Ju-Chieh (Kevin) Cheng[1,4*], Nasim Vafai[4], Elham Shahinfard[4], Carolyn English[4], Jessamyn McKenzie[5], Jing Zhang[6], Laura Barlow[7], Vesna Sossi[1]

1. Department of Physics and Astronomy, University of British Columbia, Vancouver, BC, Canada

2. Werner Siemens Imaging Center, Department of Preclinical Imaging and Radiopharmacy, Eberhard-Karls University Tuebingen, Tuebingen, Germany

3. Cluster of Excellence iFIT (EXC 2180) "Image Guided and Functionally Instructed Tumor Therapies", University of Tuebingen, Tuebingen, Germany

4. Pacific Parkinson's Research Centre, University of British Columbia, Vancouver, British Columbia, Canada

5. Djavad Mowafaghian Centre for Brain Health, Pacific Parkinson's Research Centre, University of British Columbia & Vancouver Coastal Health, Vancouver, BC, Canada

6. Global MR Applications & Workflow, GE Healthcare Canada, Vancouver, BC, Canada

7. UBC MRI Research Centre, University of British Columbia, Vancouver, British Columbia, Canada.

*authors contributed equally to the work

§corresponding author, julia.mannheim@med.uni-tuebingen.de

Methods

Phantom studies

According to NEMA specifications, RCs and %BG variability were determined using the NEMA image quality phantom (NEMA IEC Body Phantom, model PET/IEC-BODY/P, Data Spectrum Corporation, Durham, NC, USA). The phantom was scanned on the PET/MR only, as it does not fit in the HRRT FOV. The phantom is equipped with 6 fillable spheres with inner diameters of 10, 13, 17, 22, 28 and 37 mm. The two largest spheres were filled with water; the other four spheres and the phantom background volume (9800 ml) were filled with ^{18}F in a 3.88:1 contrast ratio (total activity: ~39 MBq). To simulate background activity from outside the FOV, a 70 cm fillable line inserted in a cylindrical polyethylene phantom (NEMA PET scatter phantom, model PET/NEMA-SCT/P) was filled with ~115 MBq of ^{18}F activity and positioned outside the FOV at a 5 cm distance from the image quality phantom. List-mode data were acquired for 325 s according to NEMA specifications, histogrammed into a single frame and reconstructed using TOF-OSEM with 28 subsets, 2 iterations and a 128×128×89 matrix resulting in a reconstructed voxel size of 2.781×2.781×2.780 mm^3 (reconstructed in-plane FOV: 35.6 cm, GE recommendation for phantom data). Additionally, the data were reconstructed using TOF-OSEM + 3.5 mm Gaussian transaxial and 3-point axial convolution filter (TOF + filter), TOF-OSEM + resolution modeling with point spread function (PSF, TOF + PSF) (PSF correction was implemented following the approach from (1)) and TOF-OSEM + PSF + 3.5 mm Gaussian transaxial and 3-point axial convolution filter (TOF + PSF + filter, Table 3). Attenuation correction was performed based on a manufacturer CT-generated attenuation map of the phantom; furthermore, manufacturer supplied corrections for decay, random and scattered coincidences, normalization and dead-time were applied.

Human scans

Voxel-wise correlation between activity concentration values obtained from the HRRT and PET/MR scans normalized to the injected activity were performed by placing a spherical 3D VOI encompassing the brain to investigate agreement between the two scanners over a wide range of concentration values.

Results

The measured RCs and %BG variability values of the image quality phantom (see Supplementary Figure 1 and 2 and Supplementary Table 1) were in line with values reported in literature when applying the same reconstruction parameters (2), demonstrating that the system performance is comparable to other systems from the same vendor.

Similar patterns in RCs as for the contrast phantom were observed for the image quality phantom scanned on the PET/MR (Figure 2c and Supplementary Figure 2a), although RCs were slightly lower compared to those from the contrast phantom especially for the smallest and largest hot sphere. %BG variability was significantly higher for the smallest sphere of the image quality phantom (Figure 2d and Supplementary Figure 2b).

Discussion

Differences in RCs and %BG variability between the image quality and the contrast phantom scanned on the PET/MR were observed, with lower RCs for the smallest sphere of the image quality phantom along with a larger %BG variability (Figure 2 and Supplementary Figure 2). The NEMA evaluation protocol requires a scatter phantom placed outside the FOV simulating background activity from outside the FOV. The contrast phantom data presented in the main manuscript were acquired without

this scatter phantom being in place. Hence, the increased %BG variability of the image quality phantom scanned on the PET/MR is very likely due to the background activity from the scatter phantom. Reconstruction of the image quality phantom was performed by using a 3.5mm transaxial Gaussian and a 3-point axial convolution filter, while the contrast phantom was reconstructed using a 3.5 mm Gaussian filter in all 3 dimensions to enable a direct comparison to the HRRT filtered data. Potential differences due to the two filters were evaluated and below 2.5% (data not shown). Furthermore, the image quality phantom is bigger in size, which increases attenuation, and could consequently also impact noise characteristics.

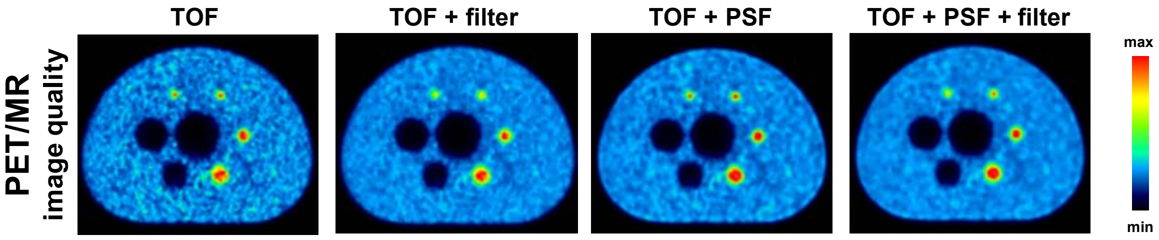

SUPPLEMENTARY FIGURE 1: Representative images of the image quality phantom data for different reconstruction parameters. Color scales of the images were normalized based on the average background value multiplied by the respective sphere to background ratio.

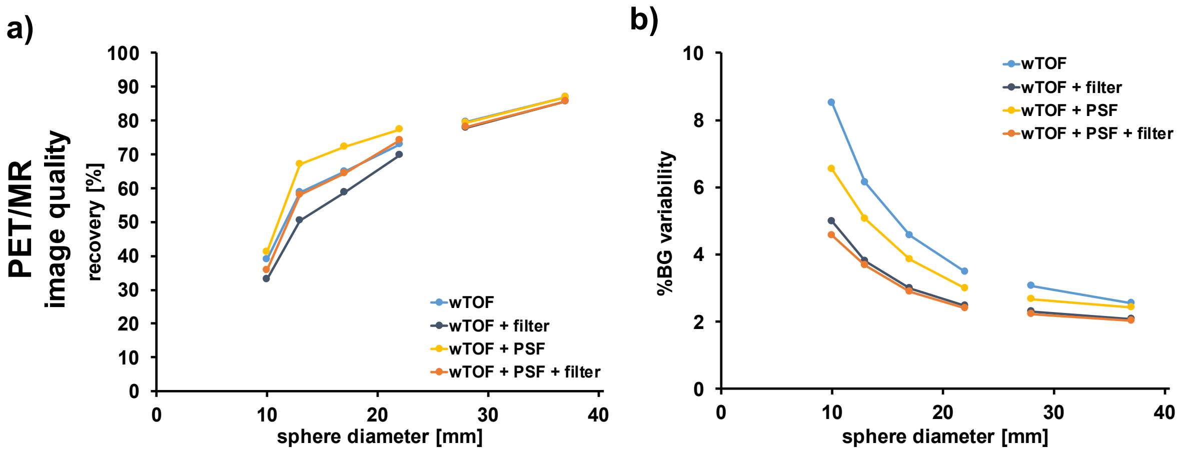

SUPPLEMENTARY FIGURE 2: RCs (a) and %BG variability (b) as a function of sphere diameter for the image quality phantom. PET/MR data was reconstructed with TOF, TOF with filter, TOF with PSF, and TOF with PSF and filter. 2 iterations and 28 subsets were used, respectively (GE recommendation for phantom data). Note the gap between spheres marks cold vs. hot spheres.

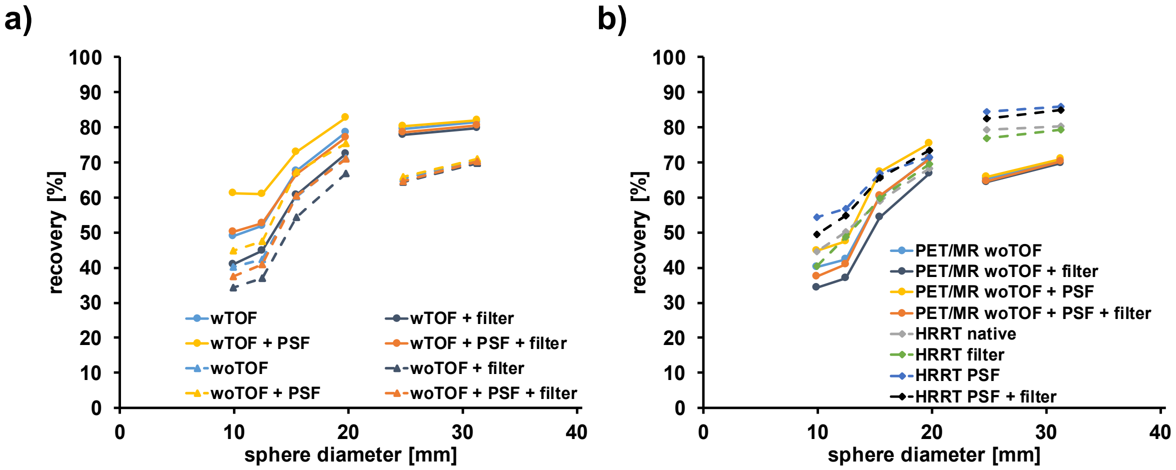

SUPPLEMENTARY FIGURE 3: RCs (%) of the PET/MR (a) as a function of sphere diameter for the contrast phantom with (solid lines) and without TOF (dotted lines). Comparison of RCs (b) without TOF of the PET/MR (solid lines) to the HRRT (dotted lines). Note the gap between spheres marks cold vs. hot spheres.

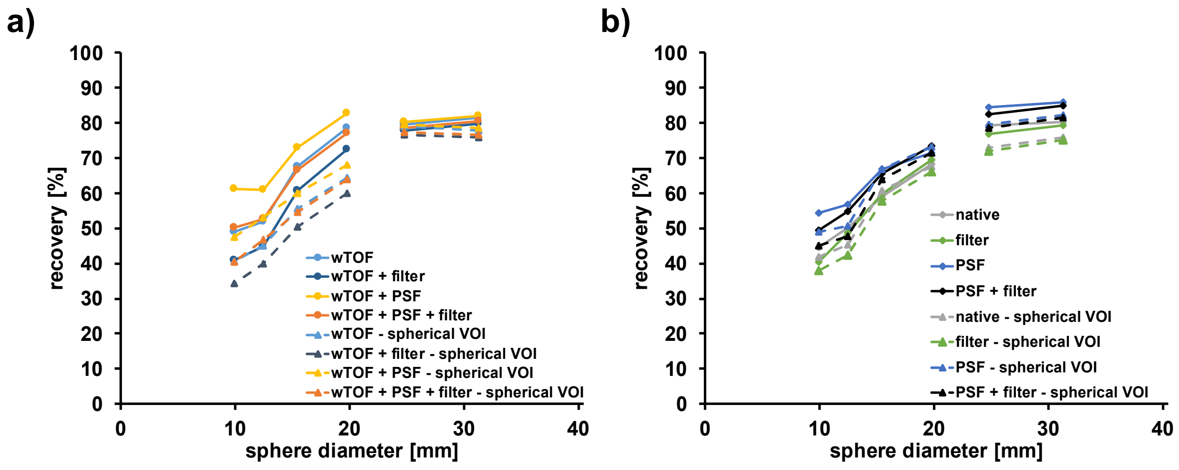

SUPPLEMENTARY FIGURE 4: RCs (%) (PET/MR (a), HRRT (b)) versus sphere diameter for the contrast phantom. Data was analyzed with a single slice ROI (solid lines), the standard NEMA analysis method, and with a spherical VOI matching the physical sphere diameter (dotted lines). Note the gap between spheres marks cold vs. hot spheres.

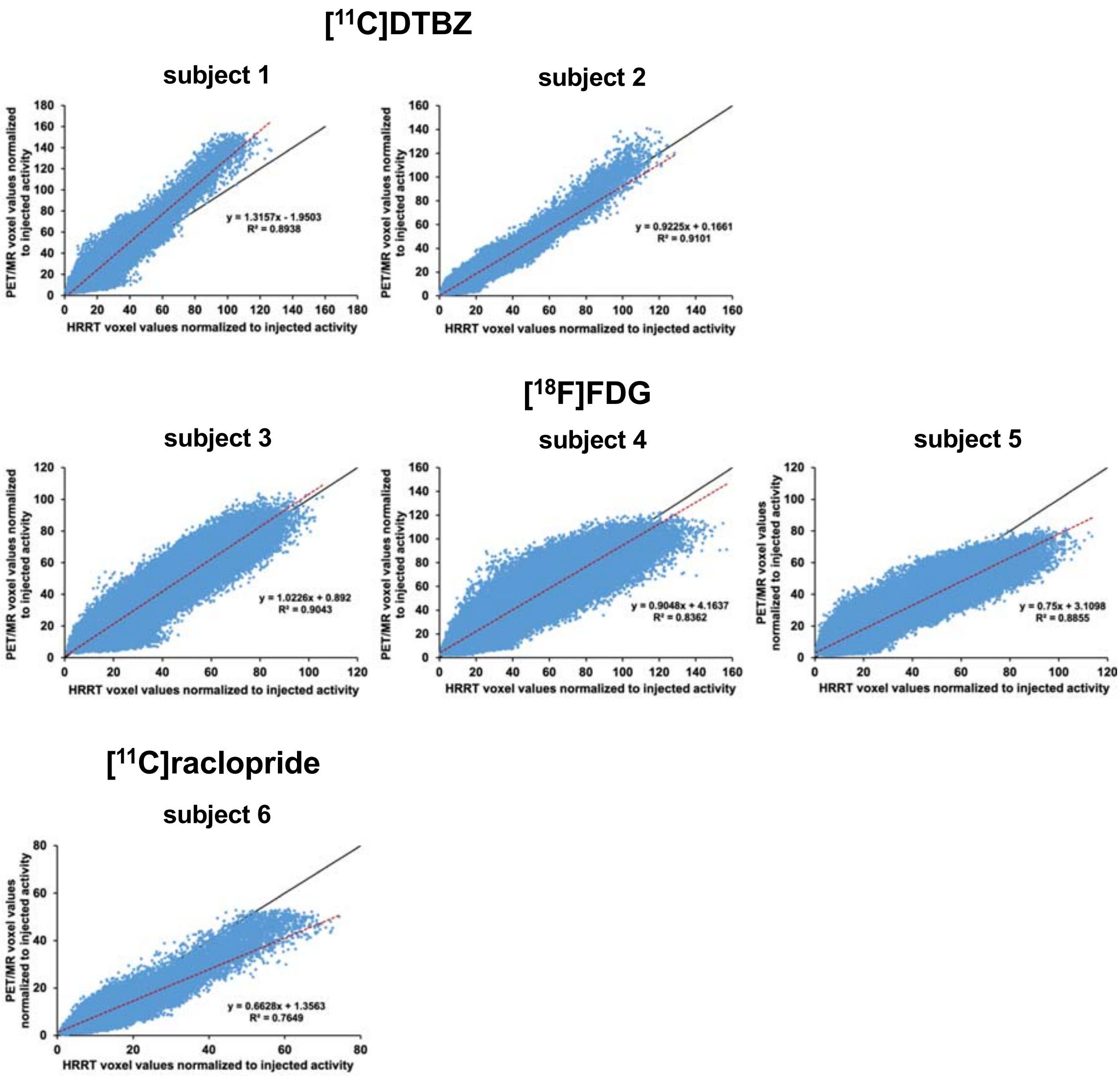

SUPPLEMENTARY FIGURE 5: Voxel-wise correlation of the HRRT to PET/MR activity concentration for each subject ([^{11}C]DTBZ, [^{18}F]FDG, [^{11}C]raclopride). The black line indicates the identity line, the red dotted line displays the linear regression of the values with the corresponding R^2.

SUPPLEMENTARY Table 1: RCs (a) and %BG variability (b) for the image quality phantom scanned on the PET/MR.

a)

			**spheres**					
			10.0 mm	13.0 mm	17.0 mm	22.0 mm	28.0 mm	37.0 mm
**PET/MR**	image quality phantom	wTOF	39.0	58.7	64.9	73.0	79.5	86.8
		wTOF + filter	33.2	50.3	58.7	69.7	77.9	85.6
		wTOF + PSF	41.2	67.1	72.2	77.5	79.3	86.9
		wTOF + PSF + filter	35.9	58.1	64.5	74.2	78.1	85.8

b)

			**spheres**					
			10.0mm	13.0mm	17.0mm	22.0mm	28.0mm	37.0mm
**PET/MR**	image quality phantom	wTOF	8.5	6.1	4.6	3.5	3.1	2.5
		wTOF + filter	5.0	3.8	3.0	2.5	2.3	2.1
		wTOF + PSF	6.5	5.1	3.9	3.0	2.7	2.4
		wTOF + PSF + filter	4.6	3.7	2.9	2.4	2.2	2.0

SUPPLEMENTARY Table 2: RCs for the contrast phantom scanned on the PET/MR and HRRT. PET/MR data were reconstructed with and without TOF information.

			spheres					
			9.9 mm	12.4 mm	15.4 mm	19.8 mm	24.8 mm	31.3 mm
**PET/MR**	wTOF	wTOF	49.0	51.9	67.7	78.5	79.5	81.4
		wTOF + filter	40.9	44.8	60.8	72.6	77.9	79.9
		wTOF + PSF	61.1	60.9	72.9	82.6	80.4	81.9
		wTOF + PSF + filter	50.2	52.6	66.7	77.2	78.7	80.5
	woTOF	woTOF	40.1	42.4	60.3	71.3	65.4	70.6
		woTOF + filter	34.3	36.9	54.4	66.7	64.3	69.8
		woTOF + PSF	44.9	47.5	67.3	75.4	65.8	71.0
		woTOF + PSF + filter	37.5	40.9	60.5	71.1	64.6	70.2
**HRRT**		native	44.5	50.1	59.1	68.4	79.3	80.4
		filter	40.4	48.7	59.6	69.4	76.8	79.3
		PSF	54.4	56.9	66.9	71.6	84.5	85.9
		PSF + filter	49.5	54.9	65.6	73.4	82.5	85.0

References

1. Alessio AM, Stearns CW, Tong S, Ross SG, Kohlmyer S, Ganin A, et al. Application and evaluation of a measured spatially variant system model for PET image reconstruction. IEEE Trans Med Imaging. 2010;29(3):938-49.

2. Grant AM, Deller TW, Khalighi MM, Maramraju SH, Delso G, Levin CS. NEMA NU 2-2012 performance studies for the SiPM-based ToF-PET component of the GE SIGNA PET/MR system. Med Phys. 2016;43(5):2334.
